# Bis[*N*,*N*-bis­(2-hy­droxy­eth­yl)glycinato]cobalt(II)

**DOI:** 10.1107/S1600536810023810

**Published:** 2010-06-26

**Authors:** Jiong-Peng Zhao, Fu-Chen Liu

**Affiliations:** a School of Chemistry and Chemical Engineering, Tianjin University of Technology, Tianjin 300191, People’s Republic of China

## Abstract

The asymmetric unit of the title compound, [Co(C_6_H_12_NO_4_)_2_], contains one half-mol­ecule with the Co^II^ ion situated on an inversion center. Inter­molecular O—H⋯O hydrogen bonds generate a three-dimensional hydrogen-bonding network, which consolidates the crystal packing.

## Related literature

For related structures, see: Ammar *et al.* (2001 ▶); Chuklanova *et al.* (1981 ▶); Thakuria & Das (2007 ▶).
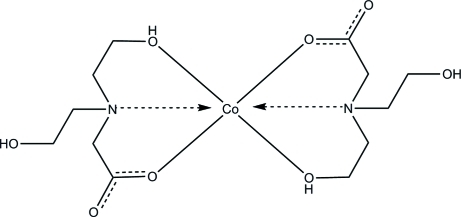


## Experimental

### 

#### Crystal data

[Co(C_6_H_12_NO_4_)_2_]
*M* *_r_* = 383.26
Monoclinic, 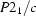

*a* = 9.932 (2) Å
*b* = 11.388 (2) Å
*c* = 7.4477 (15) Å
β = 110.12 (3)°
*V* = 791.0 (3) Å^3^
*Z* = 2
Mo *K*α radiation
μ = 1.13 mm^−1^
*T* = 293 K
0.20 × 0.18 × 0.18 mm

#### Data collection

Rigaku SCXmini diffractometer
Absorption correction: multi-scan (*ABSCOR*; Higashi, 1995 ▶) *T*_min_ = 0.736, *T*_max_ = 1.000
8129 measured reflections
1819 independent reflections
1357 reflections with *I* > 2σ(*I*)
*R*_int_ = 0.072

#### Refinement

*R*[*F* ^2^ > 2σ(*F* ^2^)] = 0.057
*wR*(*F* ^2^) = 0.159
*S* = 1.00
1819 reflections
106 parameters
H-atom parameters constrained
Δρ_max_ = 0.48 e Å^−3^
Δρ_min_ = −0.36 e Å^−3^

### 

Data collection: *SCXmini Benchtop Crystallography System Software* (Rigaku, 2006 ▶); cell refinement: *PROCESS-AUTO* (Rigaku, 1998 ▶); data reduction: *PROCESS-AUTO*; program(s) used to solve structure: *SHELXS97* (Sheldrick, 2008 ▶); program(s) used to refine structure: *SHELXL97* (Sheldrick, 2008 ▶); molecular graphics: *ORTEPIII* (Burnett & Johnson, 1996 ▶) and *PLATON* (Spek, 2009 ▶); software used to prepare material for publication: *SHELXTL* (Sheldrick, 2008 ▶).

## Supplementary Material

Crystal structure: contains datablocks global, I. DOI: 10.1107/S1600536810023810/cv2723sup1.cif

Structure factors: contains datablocks I. DOI: 10.1107/S1600536810023810/cv2723Isup2.hkl

Additional supplementary materials:  crystallographic information; 3D view; checkCIF report

## Figures and Tables

**Table 1:** Hydrogen-bond geometry (Å, °)

*D*—H⋯*A*	*D*—H	H⋯*A*	*D*⋯*A*	*D*—H⋯*A*
O2—H12⋯O3^i^	0.85	1.79	2.632 (4)	171
O1—H11⋯O3^ii^	0.85	1.89	2.744 (4)	178

Symmetry codes: (i) 
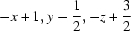
; (ii) 
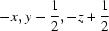
.

**Fig. 1.**
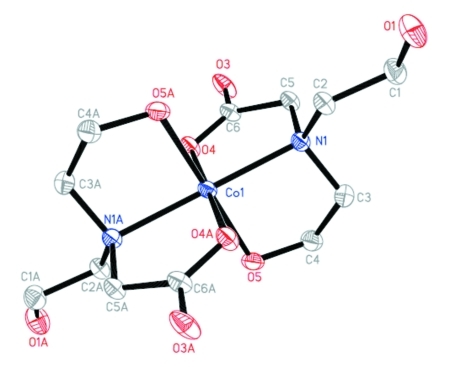
The molecular structure of the title complex. Displacement ellipsoids are drawn at the 30% probability level. H atom have been omitted for clarity. Symmetry code: (A) -x+1, -y-1, -z+1.

**Fig. 2.**
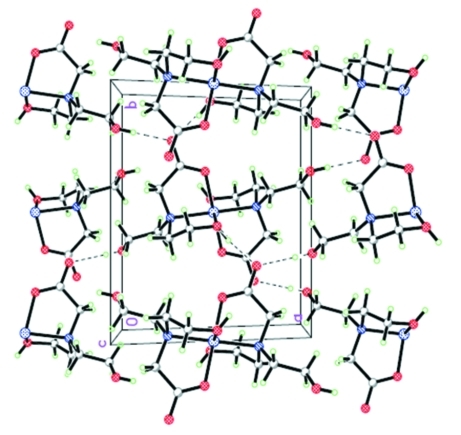
A portion of the crystal packing viewed down the c axis. Dashed lines denote O—H···O hydrogen bonds.

## supplementary crystallographic information

### Comment

As a contribution to a structural study of M*L*_2_ complexes, where *L* = N,N-bis(2-hydroxyethyl)glycinato ligand, and M = Cu (Ammar *et al.*, 2001; Thakuria & Das, 2007) and Ni (Chuklanova *et al.*, 1981), herewith we report the crystal structure of the title compound Co*L*_2_ (I).

In (I) (Fig. 1), the Co(II) ions are located on the inversion centers and are coordinated by two *L* ligands forming an octahedral enviromental geometry with four oxygen and two nitrogen atoms. The bond lengths are: Co1—N1 = 2.172 (3) Å, Co1—O2 = 2.088 (3) Å and Co1—O4 =  2.046 (2) Å. Though M*L*_2_  complexes (M = Co, Ni, Cu) have similar structures, there are some differences. The Co and Ni centers are in a regular octahedron coordinated geometry, while the Cu center has an elongated octahedral coordination with two hydroxy atoms in axial positions.

Intermolecular O—H···O hydrogen bonds (Table 1) generate three-dimensional hydrogen-bonding network, which consolidate the crystal packing (Fig. 2).

### Experimental

A mixture of Co(II) nitrate (1.0mmol), Dy(III)nitrate (0.5mmol) and N,N-bis(2-hydroxyethyl)glycine, (1 mmol), in 10 ml solvent wITH DMF:MeOH = 1:1 was sealed in a Teflon-lined stainless-steel Parr bomb that was heated at 413 K for 48 h. Red crystals of the title complex were collected after the bomb was allowed to cool to room temperature.Yield 20% based on metal salt.

### Refinement

C-bound H atoms were included in calculated positions and treated as riding on their parent atoms, with C—H = 0.93Å and *U*_iso_(H) = 1.2*U*_eq_(C). Hydroxy H atoms were located on difference Fourier maps, but placed in idealized positions (O—H = 0.85Å) and refined as riding, with *U*_iso_(H) = 1.2*U*_eq_(O).

### Crystal data

**Table:**

[Co(C_6_H_12_NO_4_)_2_]	*F*(000) = 402
*M**_r_* = 383.26	*D*_x_ = 1.609 Mg m^−^^3^
Monoclinic, *P*2_1_/*c*	Mo *K*α radiation, λ = 0.71073 Å
*a* = 9.932 (2) Å	Cell parameters from 7077 reflections
*b* = 11.388 (2) Å	θ = 3.4–27.6°
*c* = 7.4477 (15) Å	µ = 1.13 mm^−^^1^
β = 110.12 (3)°	*T* = 293 K
*V* = 791.0 (3) Å^3^	Block, red
*Z* = 2	0.2 × 0.18 × 0.18 mm

### Data collection

**Table:**

Rigaku SCXmini diffractometer	1819 independent reflections
Radiation source: fine-focus sealed tube	1357 reflections with *I* > 2σ(*I*)
graphite	*R*_int_ = 0.072
ω scans	θ_max_ = 27.5°, θ_min_ = 3.4°
Absorption correction: multi-scan (*ABSCOR*; Higashi, 1995)	*h* = −12→12
*T*_min_ = 0.736, *T*_max_ = 1.000	*k* = −14→14
8129 measured reflections	*l* = −9→9

### Refinement

**Table:**

Refinement on *F*^2^	Primary atom site location: structure-invariant direct methods
Least-squares matrix: full	Secondary atom site location: difference Fourier map
*R*[*F*^2^ > 2σ(*F*^2^)] = 0.057	Hydrogen site location: inferred from neighbouring sites
*wR*(*F*^2^) = 0.159	H-atom parameters constrained
*S* = 1.00	*w* = 1/[σ^2^(*F*_o_^2^) + (0.1*P*)^2^] where *P* = (*F*_o_^2^ + 2*F*_c_^2^)/3
1819 reflections	(Δ/σ)_max_ < 0.001
106 parameters	Δρ_max_ = 0.48 e Å^−^^3^
0 restraints	Δρ_min_ = −0.36 e Å^−^^3^

### Special details

**Table:**

Geometry. All esds (except the esd in the dihedral angle between two l.s. planes) are estimated using the full covariance matrix. The cell esds are taken into account individually in the estimation of esds in distances, angles and torsion angles; correlations between esds in cell parameters are only used when they are defined by crystal symmetry. An approximate (isotropic) treatment of cell esds is used for estimating esds involving l.s. planes.
Refinement. Refinement of *F*^2^ against ALL reflections. The weighted *R*-factor *wR* and goodness of fit *S* are based on *F*^2^, conventional *R*-factors *R* are based on *F*, with *F* set to zero for negative *F*^2^. The threshold expression of *F*^2^ > σ(*F*^2^) is used only for calculating *R*-factors(gt) *etc*. and is not relevant to the choice of reflections for refinement. *R*-factors based on *F*^2^ are statistically about twice as large as those based on *F*, and *R*- factors based on ALL data will be even larger.

### Fractional atomic coordinates and isotropic or equivalent isotropic displacement parameters (Å^2^)

**Table:**

	*x*	*y*	*z*	*U*_iso_*/*U*_eq_	
Co1	0.5000	0.5000	0.5000	0.0244 (3)	
O1	0.0043 (3)	0.3365 (3)	0.0259 (4)	0.0552 (9)	
H11	−0.0846	0.3211	−0.0073	0.066*	
O2	0.5173 (3)	0.4447 (3)	0.7743 (4)	0.0347 (7)	
H12	0.5746	0.3883	0.8218	0.042*	
O3	0.2823 (3)	0.7874 (2)	0.5700 (4)	0.0449 (8)	
O4	0.4555 (3)	0.6656 (2)	0.5703 (4)	0.0333 (6)	
N1	0.2727 (3)	0.4762 (2)	0.4479 (5)	0.0270 (7)	
C1	0.0483 (4)	0.3862 (4)	0.2103 (6)	0.0431 (10)	
H1A	0.0377	0.3299	0.3022	0.052*	
H1B	−0.0090	0.4550	0.2118	0.052*	
C2	0.2040 (4)	0.4198 (3)	0.2589 (5)	0.0329 (9)	
H2A	0.2579	0.3496	0.2538	0.040*	
H2B	0.2115	0.4730	0.1610	0.040*	
C3	0.2708 (4)	0.3967 (4)	0.6042 (6)	0.0363 (9)	
H3A	0.2918	0.3173	0.5750	0.044*	
H3B	0.1758	0.3970	0.6137	0.044*	
C4	0.3792 (4)	0.4339 (4)	0.7929 (6)	0.0401 (10)	
H4A	0.3510	0.5086	0.8317	0.048*	
H4B	0.3830	0.3762	0.8902	0.048*	
C5	0.2141 (4)	0.5937 (3)	0.4614 (6)	0.0309 (8)	
H5A	0.1554	0.5882	0.5416	0.037*	
H5B	0.1521	0.6172	0.3347	0.037*	
C6	0.3256 (4)	0.6887 (3)	0.5411 (5)	0.0310 (9)	

### Atomic displacement parameters (Å^2^)

**Table:**

	*U*^11^	*U*^22^	*U*^33^	*U*^12^	*U*^13^	*U*^23^
Co1	0.0203 (4)	0.0227 (4)	0.0279 (4)	0.0026 (3)	0.0055 (3)	−0.0003 (3)
O1	0.0397 (18)	0.066 (2)	0.053 (2)	−0.0136 (15)	0.0076 (15)	−0.0177 (16)
O2	0.0302 (14)	0.0401 (16)	0.0306 (15)	0.0078 (13)	0.0065 (11)	0.0075 (12)
O3	0.0249 (15)	0.0323 (15)	0.068 (2)	0.0053 (12)	0.0029 (14)	−0.0206 (14)
O4	0.0227 (13)	0.0269 (13)	0.0468 (17)	0.0006 (11)	0.0076 (12)	−0.0064 (11)
N1	0.0241 (16)	0.0217 (15)	0.0329 (17)	−0.0002 (12)	0.0067 (13)	−0.0023 (11)
C1	0.037 (2)	0.047 (2)	0.039 (2)	−0.013 (2)	0.0060 (18)	−0.0058 (19)
C2	0.0244 (19)	0.037 (2)	0.036 (2)	−0.0001 (16)	0.0081 (16)	−0.0030 (17)
C3	0.034 (2)	0.035 (2)	0.041 (2)	−0.0042 (18)	0.0142 (18)	0.0034 (17)
C4	0.034 (2)	0.050 (3)	0.036 (2)	0.003 (2)	0.0119 (18)	0.0074 (19)
C5	0.0216 (18)	0.034 (2)	0.035 (2)	0.0020 (16)	0.0072 (15)	−0.0028 (16)
C6	0.0258 (19)	0.032 (2)	0.031 (2)	0.0047 (16)	0.0048 (15)	−0.0049 (15)

### Geometric parameters (Å, °)

**Table:**

Co1—O4	2.046 (2)	N1—C2	1.483 (5)
Co1—O4^i^	2.046 (2)	C1—C2	1.512 (5)
Co1—O2	2.088 (3)	C1—H1A	0.9700
Co1—O2^i^	2.088 (3)	C1—H1B	0.9700
Co1—N1^i^	2.172 (3)	C2—H2A	0.9700
Co1—N1	2.172 (3)	C2—H2B	0.9700
O1—C1	1.409 (5)	C3—C4	1.507 (5)
O1—H11	0.8499	C3—H3A	0.9700
O2—C4	1.430 (5)	C3—H3B	0.9700
O2—H12	0.8500	C4—H4A	0.9700
O3—C6	1.249 (4)	C4—H4B	0.9700
O4—C6	1.260 (4)	C5—C6	1.515 (5)
N1—C5	1.476 (4)	C5—H5A	0.9700
N1—C3	1.480 (5)	C5—H5B	0.9700
			
O4—Co1—O4^i^	180.0	C2—C1—H1B	110.4
O4—Co1—O2	88.86 (11)	H1A—C1—H1B	108.6
O4^i^—Co1—O2	91.14 (11)	N1—C2—C1	115.8 (3)
O4—Co1—O2^i^	91.14 (11)	N1—C2—H2A	108.3
O4^i^—Co1—O2^i^	88.86 (11)	C1—C2—H2A	108.3
O2—Co1—O2^i^	180.0	N1—C2—H2B	108.3
O4—Co1—N1^i^	98.17 (10)	C1—C2—H2B	108.3
O4^i^—Co1—N1^i^	81.83 (10)	H2A—C2—H2B	107.4
O2—Co1—N1^i^	97.61 (11)	N1—C3—C4	111.3 (3)
O2^i^—Co1—N1^i^	82.39 (11)	N1—C3—H3A	109.4
O4—Co1—N1	81.83 (10)	C4—C3—H3A	109.4
O4^i^—Co1—N1	98.17 (10)	N1—C3—H3B	109.4
O2—Co1—N1	82.39 (11)	C4—C3—H3B	109.4
O2^i^—Co1—N1	97.61 (11)	H3A—C3—H3B	108.0
N1^i^—Co1—N1	180.0	O2—C4—C3	109.6 (3)
C1—O1—H11	108.0	O2—C4—H4A	109.8
C4—O2—Co1	111.2 (2)	C3—C4—H4A	109.8
C4—O2—H12	115.0	O2—C4—H4B	109.8
Co1—O2—H12	116.9	C3—C4—H4B	109.8
C6—O4—Co1	116.5 (2)	H4A—C4—H4B	108.2
C5—N1—C3	112.9 (3)	N1—C5—C6	114.9 (3)
C5—N1—C2	113.2 (3)	N1—C5—H5A	108.5
C3—N1—C2	110.9 (3)	C6—C5—H5A	108.5
C5—N1—Co1	106.5 (2)	N1—C5—H5B	108.5
C3—N1—Co1	103.3 (2)	C6—C5—H5B	108.5
C2—N1—Co1	109.5 (2)	H5A—C5—H5B	107.5
O1—C1—C2	106.5 (3)	O3—C6—O4	123.4 (3)
O1—C1—H1A	110.4	O3—C6—C5	117.5 (3)
C2—C1—H1A	110.4	O4—C6—C5	119.1 (3)
O1—C1—H1B	110.4		
			
O4—Co1—O2—C4	75.2 (3)	O4^i^—Co1—N1—C2	−48.4 (2)
O4^i^—Co1—O2—C4	−104.8 (3)	O2—Co1—N1—C2	−138.4 (2)
O2^i^—Co1—O2—C4	−9(84)	O2^i^—Co1—N1—C2	41.6 (2)
N1^i^—Co1—O2—C4	173.3 (3)	N1^i^—Co1—N1—C2	35 (100)
N1—Co1—O2—C4	−6.7 (3)	C5—N1—C2—C1	−67.2 (4)
O4^i^—Co1—O4—C6	37 (100)	C3—N1—C2—C1	60.8 (4)
O2—Co1—O4—C6	−88.4 (3)	Co1—N1—C2—C1	174.1 (3)
O2^i^—Co1—O4—C6	91.6 (3)	O1—C1—C2—N1	179.1 (3)
N1^i^—Co1—O4—C6	174.1 (3)	C5—N1—C3—C4	−70.5 (4)
N1—Co1—O4—C6	−5.9 (3)	C2—N1—C3—C4	161.4 (3)
O4—Co1—N1—C5	8.9 (2)	Co1—N1—C3—C4	44.2 (3)
O4^i^—Co1—N1—C5	−171.1 (2)	Co1—O2—C4—C3	32.5 (4)
O2—Co1—N1—C5	98.9 (2)	N1—C3—C4—O2	−53.5 (4)
O2^i^—Co1—N1—C5	−81.1 (2)	C3—N1—C5—C6	101.7 (4)
N1^i^—Co1—N1—C5	−88 (100)	C2—N1—C5—C6	−131.4 (3)
O4—Co1—N1—C3	−110.2 (2)	Co1—N1—C5—C6	−11.0 (4)
O4^i^—Co1—N1—C3	69.8 (2)	Co1—O4—C6—O3	−177.0 (3)
O2—Co1—N1—C3	−20.3 (2)	Co1—O4—C6—C5	1.1 (5)
O2^i^—Co1—N1—C3	159.7 (2)	N1—C5—C6—O3	−174.3 (3)
N1^i^—Co1—N1—C3	153 (100)	N1—C5—C6—O4	7.4 (5)
O4—Co1—N1—C2	131.6 (2)		

Symmetry codes: (i) −*x*+1, −*y*+1, −*z*+1.

### Hydrogen-bond geometry (Å, °)

**Table:**

*D*—H···*A*	*D*—H	H···*A*	*D*···*A*	*D*—H···*A*
O2—H12···O3^ii^	0.85	1.79	2.632 (4)	171
O1—H11···O3^iii^	0.85	1.89	2.744 (4)	178

Symmetry codes: (ii) −*x*+1, *y*−1/2, −*z*+3/2; (iii) −*x*, *y*−1/2, −*z*+1/2.
